# Five Questions about Viruses and MicroRNAs

**DOI:** 10.1371/journal.ppat.1000787

**Published:** 2010-02-26

**Authors:** Bryan R. Cullen

**Affiliations:** Department of Molecular Genetics & Microbiology and Center for Virology, Duke University Medical Center, Durham, North Carolina, United States of America; University of California San Francisco, United States of America

MicroRNAs (miRNAs) are ∼22-nt regulatory RNAs expressed by all multicellular eukaryotes [1]. Humans encode >700 miRNAs and similar numbers are likely to exist in other mammalian species. Almost all cellular miRNAs are initially transcribed by RNA polymerase II (Pol II) as part of a long, capped, polyadenylated primary miRNA (pri-miRNA) precursor. The miRNA forms part of one arm of an RNA stem-loop that consists of an ∼32-bp imperfect stem flanked by unstructured RNA sequences. This stem-loop is recognized by the nuclear RNase III enzyme Drosha, which cleaves the stem to liberate an ∼60-nt pre-miRNA hairpin. The pre-miRNA is then transported to the cytoplasm where it is cleaved by a second RNase III enzyme, called Dicer, which removes the terminal loop to generate the miRNA duplex intermediate. One strand of this duplex is incorporated into the RNA-induced silencing complex (RISC), where it acts as a guide RNA to direct RISC to complementary mRNA species [1]. Depending on the level of complementarity, RISC can either cleave bound mRNAs and/or inhibit their translation. Inhibition of mRNA translation generally requires full complementarity of the mRNA to nucleotides 2 through 7 or 8 from the miRNA 5′ end—the miRNA seed region. The primary, and possibly sole, function of mammalian miRNAs is therefore to act as specific post-transcriptional inhibitors of mRNA function.

## Do All Viruses Encode miRNAs?

Because miRNAs are potent regulators of gene expression that are both small and non-antigenic, they would seem ideal tools to alter the cellular environment in ways that favor virus replication. While numerous virally encoded miRNAs have, in fact, been described for members of the herpesvirus family, few other viral miRNAs have been identified (Table 1). In general, we can divide mammalian viruses into three categories, i.e., the herpesviruses, which encode multiple viral miRNAs; other nuclear DNA viruses, which may encode one or two miRNAs; and the RNA viruses and cytoplasmic DNA viruses, which appear to lack any miRNAs [1]. While this generalization is clearly subject to revision as more viruses are analyzed, viruses that currently appear to lack any miRNAs include retroviruses (e.g., human immunodeficiency virus type 1 [HIV-1]), flaviviruses (e.g., yellow fever virus), hepatitis C virus (HCV), and papillomavirus [2],[3]. In addition, deep sequencing of small RNAs isolated from cells infected with cowpox virus or influenza A virus has also failed to identify any virally encoded miRNAs (J. L. Umbach and B. R. Cullen, unpublished data).

**Table 1:** MicroRNAs Encoded by Mammalian Viruses.

Virus Family	Virus Species	Host	Known miRNAs
α-herpesviruses	HSV-1	Human	8
	HSV-2	Human	6
β-herpesviruses	hCMV	Human	11
	mCMV	Murine	18
γ-herpesviruses	EBV	Human	25
	rLCV	Simian	33
	KSHV	Human	12
	RRV	Simian	15
	MHV68	Murine	9
Polyomaviruses	polyoma	Murine	1
	SV40	Simian	1
	JCV	Human	1
	BKV	Human	1
Adenovirus	hAV	Human	2

This table lists the number of currently known pre-miRNA stem-loops encoded by each virus listed. BKV, BK virus; hAV, human adenovirus; JCV, JC virus; mCMV, murine cytomegalovirus; MHV68, mouse γ-herpesvirus 68.

Although the reason for this pattern of viral miRNA expression remains unknown, one can speculate that viruses that replicate in the cytoplasm (e.g., flaviviruses, HCV, and cowpox) might lack miRNAs because they never encounter the nuclear enzyme Drosha. On the other hand, nuclear RNA viruses (e.g., influenza and HIV-1) may avoid encoding miRNAs because excision of a genomically encoded pre-miRNA would induce the cleavage and degradation of the viral RNA genome. Finally, one can speculate that viruses that establish long-term, latent infections (e.g., herpesviruses) would derive more benefit from the use of non-antigenic miRNAs to inhibit cellular antiviral responses than would viruses that have short lytic replication cycles.

## What Do Viral miRNAs Do?

Only a small number of mRNA targets of viral miRNAs have been defined, so a complete answer to this question is currently not possible. However, the emerging picture [4] suggests that viral miRNAs may serve two major functions. On the one hand, several viral miRNAs have been shown to inhibit the expression of cellular factors that play a role in cellular innate or adaptive antiviral immune responses. Examples of the inhibition of host cell factors involved in mediating immune responses include the natural killer cell ligand MICB, which is downregulated by miRNAs encoded by human cytomegalovirus (hCMV), Kaposi's sarcoma-associated herpesvirus (KSHV), and Epstein-Barr virus (EBV) [5], and the pro-apoptotic protein PUMA, which is downregulated by an EBV miRNA [6]. The potential selective advantage conferred on a virus by this downregulation is obvious. Secondly, several viral miRNAs downregulate the expression of viral proteins, including key viral immediate-early or early regulatory proteins. For example, herpes simplex virus 1 (HSV-1) downregulates the immediate-early transactivators ICP0 and ICP4 in latently infected cells by using viral miRNAs that are expressed at high levels during latency, but not during productive viral replication. This action is thought to stabilize the latent state [7]. Conversely, several polyomaviruses downregulate the expression of the viral T antigens, key early transcription factors, at late stages in the viral replication cycle [8],[9],[10]. It has been proposed that this reduces the killing of infected cells by cytotoxic T lymphocytes specific for T antigen epitopes [8].

Although it is therefore widely believed that viral miRNAs play an important role in the replication and pathogenesis of the viruses that encode them, little evidence in support of this hypothesis currently exists. Indeed, a mutant polyomavirus lacking the only polyomavirus-encoded miRNA replicated indistinguishably from wild-type in infected mice [9]. Whether herpesviruses, which encode more viral miRNAs, do depend on these small RNAs will no doubt soon be revealed.

## Are Viral miRNAs Conserved between Related Viral Species?

If viral miRNAs do play an important role in viral replication and pathogenesis, then they should be under purifying selection. Indeed, comparison of the genomic sequences of diverse KSHV isolates reveals that the miRNAs encoded by KSHV are, in general, highly conserved, although isolates bearing mutations that block production of individual KSHV miRNAs have been described [11]. A similar high level of conservation also appears to be characteristic of miRNAs encoded by other human herpesviruses, including EBV and hCMV.

In contrast, a comparison between distinct but related herpesvirus species reveals far less evidence of miRNA sequence conservation, although the genomic localization of virally encoded miRNAs is often conserved. In the case of HSV-1, which encodes eight known viral miRNAs, and the related but distinct HSV-2, which encodes at least six miRNAs, four miRNAs are partially conserved in terms of sequence, and fully conserved in terms of genomic localization [12]. Similarly, 22 of the 25 miRNAs encoded by EBV are at least partially conserved in the related γ-herpesvirus rhesus lymphocryptovirus (rLCV), which encodes a remarkable 33 miRNAs [13]. In contrast, none of the 12 miRNAs expressed by KSHV are conserved in the somewhat more distantly related rhesus rhadinovirus (RRV), although all virally encoded miRNAs are found in a single cluster located adjacent to the viral *ORF71* gene in both virus species [2],[13],[14]. It is therefore likely that viral miRNAs evolve through a combination of sequence evolution of pre-existing miRNA stem-loops combined with an occasional stem-loop duplication event [13]. As miRNAs are short, readily mutable sequences, any degree of evolutionary conservation implies that miRNAs can confer a significant selective advantage. Presumably, sequence conservation of viral miRNAs also implies the existence of cellular, rather than viral, mRNA targets, as the latter might be expected to rapidly co-evolve with viral miRNAs.

## Do Viruses Regulate miRNA Transcription, Processing, or Function?

Viral infection represents a cataclysmic event in the life of a cell that generally results in dramatic changes in cellular mRNA expression. These may include the induction of cellular factors that have the potential to resist or promote viral replication. As cellular miRNAs are also transcribed by Pol II, it is expected that viral infection would also affect their pattern of expression. The question then is whether these changes are evolutionarily selected to favor or inhibit virus replication. At present, there is no evidence indicating that viruses modify the cellular environment to selectively favor the processing and expression of viral miRNAs, although viral miRNAs may be expressed at such high levels that they have the potential to competitively inhibit cellular miRNA function.

While it has been proposed that HIV-1 infection induces the expression of specific cellular miRNAs [15], the best-characterized example of a virus inducing cellular miRNAs that may favor virus infection occurs in the case of EBV, which induces the expression of several cellular miRNAs, including miR-155, in infected B cells [16]. In contrast, while KSHV does not induce miR-155 expression upon infection of B cells, it does encode a viral miRNA that functions as an ortholog of miR-155 [17],[18]. As miR-155 can promote the oncogenic transformation of B cells when overexpressed in vivo, it is likely that miR-155 induction by EBV, or expression of a miR-155 mimic by KSHV, plays a role not only in promoting the establishment of EBV or KSHV latency, but also in viral transformation of B cells [16],[17],[18].

While viruses may therefore enhance cellular miRNA expression, the more commonly observed effect is inhibition [15],[19]. Moreover, there have been several reports demonstrating that cellular miRNAs can inhibit virus replication and/or that artificial inhibition of miRNA production or function can enhance virus replication in culture [15],[20],[21],[22],[23]. Many viruses globally repress Pol II transcription in infected cells, and this would also repress miRNA biogenesis. In addition, adenoviruses produce the viral VA1 non-coding RNA, which acts as a potent inhibitor of both pre-miRNA nuclear export and pre-miRNA processing by Dicer [24]. The HIV-1 Tat protein has also been proposed to inhibit miRNA function, although this appears to require very high levels of Tat expression [19].

## Can Viruses Use Cellular miRNAs to Promote Their Replication?

At least one clear-cut example of a cellular miRNA that facilitates virus replication is known, i.e., activation of HCV replication by the liver-specific miRNA miR-122 [25]. The HCV RNA genome contains two miR-122 binding sites in its 5′ untranslated region that are required for activation of viral genomic RNA replication, although the underlying mechanism remains obscure. At present, this result remains unique to HCV; i.e., no other RNA virus has been shown to rely upon direct binding of a cellular miRNA to its RNA genome for any aspect of its replication cycle.

An interesting and largely unresolved aspect of the replication cycle of RNA viruses is that their RNA genome must serve several essential yet mutually exclusive roles. Thus, the HCV genome is not only the mRNA that encodes all the viral proteins but also the substrate for the viral RNA-dependent RNA polymerase and the genomic RNA that is packaged into progeny virions. It is interesting to speculate that cellular miRNAs could play a role in retargeting viral RNAs away from translation and towards one of the latter two fates. Of note, RISC is believed to direct bound mRNAs into cytoplasmic processing bodies (P bodies), where they may be translationally repressed and/or degraded, yet emerging evidence suggests that P bodies can also function as sites of viral RNA synthesis [26]. Currently, it remains unclear how viruses deal with the potentially inhibitory effect of the large numbers of cellular miRNAs that have at least the potential to target viral RNA genomes and/or mRNAs. Have they simply evolved to inhibit miRNA function and/or minimize the number of functional binding sites for the miRNA species that are expressed in infected cells, or have they instead evolved ways to take advantage of these miRNAs? This issue is one that deserves significant attention, as it may suggest novel methods to selectively perturb the replication of pathogenic viruses in vivo.
